# Corneal endothelial cell loss after trabeculectomy and phacoemulsification in one or two steps: a prospective study

**DOI:** 10.1038/s41433-020-01331-x

**Published:** 2021-01-07

**Authors:** María Isabel Soro-Martínez, Juan Antonio Miralles de Imperial-Ollero, Miriam Pastor-Montoro, Gabriel Arcos-Villegas, Paloma Sobrado-Calvo, José María Ruiz-Gómez, Jaime Miralles de Imperial-Mora-Figueroa, María Paz Villegas-Pérez

**Affiliations:** 1 Ophthalmology Service, Hospital General Universitario Reina Sofía, Avda Intendente Jorge Palacios s/n, 30003 Murcia, Spain; 2 Ophthalmology Service, Fundación Jiménez Díaz University Hospital, Avda de los Reyes Católicos 2, 28040 Madrid, Spain; 3 Ophthalmology Departament, Edificio Departamental, Campus de Ciencias de la Salud, University of Murcia, Carretera a Buenavista s/n, 30120 El Palmar, Murcia Spain; 4 Statistics and Operational Research Departament, University of Murcia, Campus de Espinardo, 30100 Espinardo, Murcia Spain

**Keywords:** Glaucoma, Outcomes research

## Abstract

**Objective:**

The objective of this study was to analyse the results of the surgical treatment of coexisting cataract and glaucoma and its effects on corneal endothelial cell density (CECD).

**Methods:**

We include two longitudinal prospective studies: one randomised that included 40 eyes with open angle glaucoma that received one- (*n* = 20) or two-step (*n* = 20) phacotrabeculectomy and another that included 20 eyes that received phacoemulsification. We assess the impact of surgery on different clinical variables and in particular in CECD using Confoscan 4™ confocal microscopy and semiautomatic counting methods.

**Results:**

Phacoemulsification and phacotrabeculectomy, but not trabeculectomy, increase significantly best-corrected visual acuity and anterior chamber depth and trabeculectomy and one- or two-step phacotrabeculectomy decreased similarly the intraocular pressure. We document percentages of endothelial cell loss of 3.1%, 17.9%, 31.6% and 42.6% after trabeculectomy, phacoemulsification and one- or two-step phacotrabeculectomy, respectively. The coefficient of variation did not increase significantly after surgery but the percentage of hexagonality decreased significantly after phacoemulsification and after two-step phacotrabeculectomy.

**Conclusions:**

Trabeculectomy, phacoemulsification and phacotrabeculectomy are surgical techniques that cause morphological changes and decrease the densities of the corneal endothelial cells. Trabeculectomy produces lesser endothelial cell loss than phacoemulsification, and phacoemulsification lesser cell loss than phacotrabeculectomy. Two-step phacotrabeculectomy (trabeculectomy followed 3 months later by phacoemulsification) causes more cell loss than one-step phacotrabeculectomy, and this could be due to the cumulative effects of two separate surgical traumas or to a negative conditioning lesion effect of the first surgery. For the treatment of coexisting glaucoma and cataract, one-step phacotrabeculectomy is the treatment of choice.

## Introduction

Corneal transparency is critical for vision and depends largely on the function of the corneal endothelial cells. These cells have a regular hexagonal form in children, but with age they show an increase in size (polymegathism) and a decrease of their regular hexagonal morphology (pleomorphism) [1–3]. Corneal endothelial cell density (CECD) is normally between 2600 and 2900 cells/mm^2^ in adults [3–5] and decreases in corneal diseases or after anterior segment surgery. When CECD is below 400–700 cells/mm^2^, there is corneal oedema and vision loss. At present, the most common aetiology of corneal oedema is cataract surgery [6]. Filtering glaucoma surgery causes less cell loss than cataract surgery [7–16], but it may also cause significant cell loss, especially if combined with cataract surgery [12, 14, 17, 18].

In a previous study [14], we analysed retrospectively the CECD after one- or two-step phacotrabeculectomy that had been performed between 6 months and 5 years before and we documented percentages of cell loss of 6.35%, 24.85% and 40.78% after trabeculectomy, or one- or two-step phacotrabeculectomy, respectively. In this article, we present the findings of two different prospective studies in which we have analysed CECD after one- or two-step phacotrabeculectomy and after phacoemulsification.

## Methods

Two longitudinal prospective cohort studies are included that were carried out sequentially. In the first, we analyse the impact of one- or two-step phacotrabeculectomy on CECD and other clinical parameters after 1, 3, 6 and 12 months. Because in this study we documented that corneal endothelial cell loss occurs in the 1st month after surgery, in the second study, we analyse the impact of phacoemulsification alone on CECD 1 month after surgery. The two studies were approved by the Clinical Ethics Committee of our hospital and followed the ethical principles for medical research of the Declaration of Helsinki. The size of the groups was 20 patients in each subgroup/group assessed for normality because that was the size used in our previous study [14] that allowed us to discriminate small CECD losses.

### Short- and long-term prospective study: one- or two-step phacotrabeculectomy

This longitudinal prospective study was randomised, controlled, double-blind and had two arms. The inclusion criteria were: (1) age > 40 years; (2) diagnosis of open angle glaucoma and cataract; (3) best-corrected visual acuity (BCVA) ≤ 0.6; (4) intraocular pressure (IOP) corrected by central corneal thickness (CCT) [19] after washout > 26 mm Hg. The exclusion criteria were: (1) previous intraocular surgery; (2) uveitic, traumatic, neovascular or closed angle glaucoma; (3) ocular pathologies other than glaucoma or cataract.

The patients selected interrupted the topical hypotensive treatment and were appointed for the baseline visit 1 month later. Forty eyes of 38 patients were included and randomised (random numbers 1 and 2) to receive phacotrabeculectomy in one step (group 1) or two steps (group 2: trabeculectomy, followed 3 months later by phacoemulsification). When corrected IOP ≥ 22 mm Hg after surgery, topical hypotensive treatment was re-started.

In the one-step group (group 1), eyes were examined at baseline and 1, 3, 6 and 12 months after phacotrabeculectomy. In the two-step group (trabeculectomy and 3 months later phacoemulsification, group 2), eyes were examined at baseline, 1, 2 and 3 months after trabeculectomy and 1, 3, 6 and 12 months after phacoemulsification. At each visit, we determined BCVA (Snellen charts), IOP by applanation tonometry, CCT (Ocuscan® RxP, Alcon Inc., Irvine, CA, USA) and anterior chamber depth (ACD; IOL Master® ver. 4; Carl Zeiss, Meditec, Germany), and performed Confoscan 4™ confocal corneal microscopy (see below).

### Short-term prospective study: cataract surgery only

The prospective study was randomised, controlled, double-blind with only one arm. The inclusion criteria were: inclusion in the waiting list for cataract surgery and the inclusion criteria numbers 1 and 3 of the previous group. The exclusion criteria were the same as in the previous group and, in addition, glaucoma. The BCVA and Confoscan 4™ corneal biomicroscopy were performed 1 week before surgery and 1 month after surgery.

### Confocal endothelial cell biomicroscopy

**Fig. 1:**
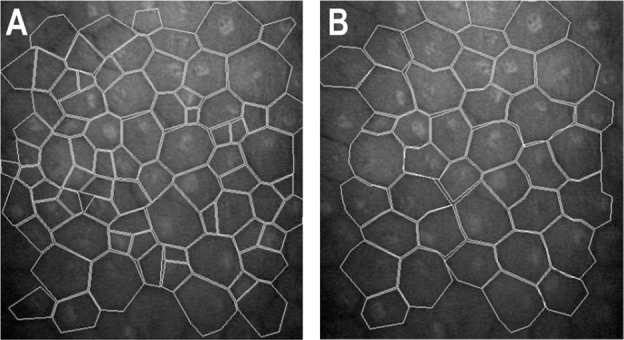
Confoscan 4™ corneal endothelial cell counting. Images corresponding to the automatic (**A**) and semiautomatic (**B**) methods of identification of the endothelial cells with the Confoscan 4™ in a patient included in the study that had densities of 1617 cells/mm^2^ with the automatic method (**A**) and of 886 cells/mm^2^ with the semiautomatic method.

Confocal corneal biomicroscopy was done with Confoscan 4™ (Nidek, Tokyo, Japan), and four images (AOI; 460 × 345 µm) from the corneal endothelium were selected for each eye at each study period and analysed using the Confoscan software Navis®. We performed an automatic and a semiautomatic analysis using manual identification of the cells, and the number of cells included in the AOI was always ≥35. Because our qualitative observations indicated that the automatic counting overestimated CECD (Fig. 1), we used the semiautomatic method primarily.

### Surgical technique

Cataract and/or glaucoma surgery in the first study were carried out by two experienced surgeons (JMIMF and MPVP) and cataract surgery in the second study only by one surgeon (MPVP). The technique for trabeculectomy was a “macrotrabeculectomy” [10, 14, 20] derived from the Watson technique [21] and comprised a limbus-based conjunctival flap, a 8 × 5 mm superficial scleral flap and a 4 × 4 deep scleral flap as described in our previous article [14]. Antimitotics were not used during or after surgery because we do not use them routinely in absence of failure risk factors. Phacoemulsification (AMO White Star™) was done through a 2.75 mm corneal incision and the intraocular acrylic lens implanted in the capsular sac were either and Acrysof® SN60 (Alcon, Fort Worth, TX, Novartis) or the AMO Sensar® AR40 (AMO Inc., Santa Ana, CA, USA) lenses. Viscoelastics: Healon Endocoat® OVD and Healon® Pro were used during phacoemulsification. At the end of surgery, we administered 0.1 ml of 0.1% cefuroxime (or 0.1% vancomycin, in penicillin-allergic patients) intracamerally. Postoperative treatment included drops containing 3% tobramycine and 1% dexamethasone (Tobradex®, Alcon) in tapering dose for 5 weeks and an unguent containing Prednisone-neomycine® at night (Alcon). After trabeculectomy, the patients received in addition ciclopentolate 10% eyedrops (Colirio ciclopléjico®, Alcon) for 1 month and oral ibuprofen 400–600 mg every 8 h during the 1st week. Eyes with surgical complications other than transitory corneal oedema, hyphema, hypotony or mild choroidal detachment were not included in the study.

### Data analysis

Data was analysed using the SPSS 15.0 programme and/or the GraphPad Prism programme. Normality was assessed using the Kolmogorov–Smirnov test, and comparisons between groups were done with the one-way ANOVA followed by Tukey’s (parametric) or the Kruskal–Wallis followed by the Mann–Whitney (non-parametric) tests. To compare variables at different intervals within the same group we used the Student’s *t* test or the Mann–Whitney test. Qualitative values were compared using the chi-square.

## Results

### Prospective study: phacotrabeculectomy in one or two steps

Forty eyes of 38 Caucasian patients (18 male and 20 females) were included, 20 in each one- and two-step groups (Table 1). There were no significant differences in the age or gender of the patients between the groups. Three eyes in group 1 and six eyes in group 2 had pseudoexfoliative glaucoma, but this difference was not statistically significant. All the other eyes had primary open angle glaucoma.

**Table 1:** Variables of the two prospective studies and comparisons between them.

		Phacotrabeculectomy			Phacoemulsification	Comparison between the two studies
	Months	Group 1: one step, *n* = 20	Group 2: two steps, *n* = 20	Comparison between groups 1 and 2 (first study)	*n* = 20	
Gender		10M/10F	8M/12F	**p = 0.58, chi-square	11M/9F	**p = 0.626, chi-square
Age ± SD		74.05 ± 5.22	72.25 ± 7.37	**p = 0.379, Tukey’s	70.35 ± 6.21	**p = 0.079, Kruskal–Wallis
Pseudoexfoliation (*n*=)		3/20	6/20	**p = 0.451, chi-square		
CCT	Baseline	529 ± 41	534 ± 36	**p = 0.499, Mann–Whitney		
Central CECD	Baseline	2558 ± 309	2485 ± 388	*p* = 0.517, Student’s *t*	2528 ± 383	**p = 0.817, one-way ANOVA
CECD after trabeculectomy only (% loss)	1		2424 ± 440^b^ (**2.5%**)			
	3		2408 ± 415^b^ (**3.2%**)			
CECD after phacotrabeculectomy (% loss)	1	1772 ± 484^a^ (**30.7%**)	1402 ± 586^a^ (**43.6%**)	*p = 0.036, Student’s *t*	2066 ± 434^a^ (**17.9%**)	*p < 0.001, one-way ANOVA
	3	1736 ± 506^a,c^	1369 ± 525^a,c^	*p = 0.030, Student’s *t*		
	6	1785 ± 480^a,c^	1417 ± 516^a,c^	*p = 0.025, Student’s *t*		
	12	1769 ± 509^a,c^ (**31.62%**)	1396 ± 514^a,c^ (**42.55%**)	*p = 0.027, Student’s *t*		
Polymegathism (COV)	Baseline	38.5 ± 10.9	40.0 ± 10.1	**p = 0.989, Mann–Whitney	40.2 ± 6.6%	**p = 0.309, Kruskal–Wallis
COV after phacotrabeculectomy	1	32.4 ± 6.3^b^	35.5 ± 10.9^b^	**p = 0.525, Mann–Whitney	43.6 ± 6.4%^b^	*p < 0.001, Kruskal–Wallis
	12	34.2 ± 4.2^b^	34.9 ± 8.5^b^	**p = 0.797, Mann–Whitney		
Pleomorphism	Baseline	54.1 ± 8.2	52.7 ± 8.5	**p = 0.543, Mann–Whitney;	50.6 ± 7.4	**p = 0.403, one-way ANOVA
	1	45.6 ± 14.7^b^	35.9 ± 10.4^a^	*p = 0.022, Mann–Whitney	43.3 ± 7.3^a^	*p = 0.022, one-way ANOVA
	12	48.8 ± 12.3^b,c^	39.6 ± 10.9^a,c^	*p = 0.009, Mann–Whitney		

*M* male, *F* female, *SD* standard deviation, *CCT* central corneal thickness, *CECD* corneal endothelial cell density.

*Significant differences between groups 1 and 2 of the first study or between groups of the first and second study.

**Not significant differences between groups 1 and 2 of the first study or between groups of the first and second study.

^a^Significant differences with preoperative values.

^b^Not significant differences with preoperative values.

^c^Not significant differences with previous postoperative value.

The bold values are the observed percentajes of corneal endothelial cell loss in the different groups/subgroups.

#### Best-corrected visual acuity

Preoperative BCVA was similar in both groups (0.47 ± 0.18 and 0.40 ± 0.17 in groups 1 and 2, respectively; Mann–Whitney, *p* = 0.181). In group 1 (one step), BCVA increased significantly to 0.84 ± 0.2 in the 1st month after surgery (Kruskal–Wallis, *p* = 0.000), but not thereafter (Mann–Whitney, *p* ≥ 0.134). In group 2 (two step), BCVA did not increase significantly after trabeculectomy (Kruskal–Wallis, *p* = 0.996), increased significantly to 0.87 ± 0.20 in the 1st month after phacoemulsification (Kruskal–Wallis, *p* = 0.000) but not thereafter (Mann–Whitney, *p* ≥ 0.141). There were no differences in BCVA between the groups at any postoperative period (Mann–Whitney, *p* ≥ 0.218).

#### Topical treatment

The mean number of topical hypotensive drugs was similar in both groups at baseline (1.21 ± 0.55 and 1.26 ± 0.42 in groups 1 and 2, respectively; range: 0–2, *p* = 0.678; chi-square) and also 12 months after surgery (0.20 ± 0.43 and 0.25 ± 0.46 in groups 1 and 2, respectively; *p* = 0.704; chi-square). Four eyes required treatment in the one-step group and two eyes required treatment in the two-step group.

#### Intraocular pressure

Mean baseline IOP after washout was 29 ± 4 and 33 ± 5 in groups 1 and 2, being significantly higher in the two-step group (Mann–Whitney, *p* = 0.025). In the one-step group, mean IOP decreased immediately to 14 ± 3 in the 1st month after surgery and remained stable thereafter (Mann–Whitney, *p* ≥ 0.275). In the two-step group, mean IOP decreased significantly to 15 ± 5 in the 1st month after trabeculectomy and did not vary significantly thereafter or after cataract surgery (Kruskal–Wallis, *p* = 0.801). When we compared the IOP at between the one-step and two-step groups, there were no significant differences at any postoperative period (Mann–Whitney, *p* > 0.208).

#### Central corneal thickness

The CCT was similar in the one- and two-step groups at baseline (Table 1) and at all the postoperative periods (not shown; Mann–Whitney, *p* > 0.218). Baseline CCT did not show significant variations postoperatively within the groups (Kruskal–Wallis, *p* = 0.920 for group 1 and *p* = 0.614 for group 2).

#### Anterior chamber depth

Mean ACD was 2.84 ± 0.43 and 3.10 ± 0.30 in groups 1 and 2 at baseline and 4.01 ± 0.49 and 4.46 ± 0.44 at 12 months after surgery and was thus significantly lower at baseline and 6 and 12 months after phacotrabeculectomy in the one-step group (*p* < 0.048; Mann–Whitney). ACD did not increase significantly after trabeculectomy (Kruskal–Wallis, *p* = 0.747) in the two-step group but increased significantly after cataract surgery in both groups.

#### Central corneal endothelial cell density

We analysed 57.94 ± 21.75 cells per image (range: 35–212). Baseline CECD was similar in the one- and two-step groups (Table 1). CECD decreased significantly in the 1st month after surgery in the one-step group (Tukey, *p* < 0.000; Table 1) but later remained stable (Tukey, *p* > 0.754). In the two-step group, CECD did not decrease significantly after trabeculectomy (ANOVA, *p* = 0.825; Table 1) but decreased significantly in the 1st month after cataract surgery (Tukey, *p* < 0.000) and remained stable thereafter (Tukey, *p* > 0.772). When we compared CECD between the groups, we found significantly higher cell densities in the one-step group at all the postoperative intervals (Table 1). Twelve months after surgery, the percentages of cell loss amounted 31.6% in the one-step group and 42.6% in the two-step group.

Because the numbers of patients with pseudoexfoliation were three and six in the one- and two-step groups, respectively, and this type of glaucoma affects adversely the corneal endothelium, we analysed the data excluding these patients. Twelve months after surgery, CECD excluding pseudoexfoliation patients were 1663 ± 438 and 1463 ± 528 in the one-step and two-step groups, and these densities were not significantly different (*t*-test, *p* = 0.2675).

#### Polymegathism and pleomorphism

The COV or polymegathism decreased non-significantly after one- or two-step surgery (Table 1; Kruskal–Wallis, *p* = 0.161 and *p* = 0.238, respectively). The percentage of hexagonality decreased non-significantly in the one-step group (Kruskal–Wallis, *p* = 0.238) and significantly in the two-step group in the 1st month after cataract surgery (Mann–Whitney, *p* < 0.000), although it did not decrease significantly thereafter (Mann–Whitney, *p* > 0.283). When we compared the COV and the percentage of hexagonality between the groups, we found that the COV was similar at all periods, but the pleomorphism was significantly lower in the two-step group at all the postoperative periods (Table 1).

### Short-term prospective study: cataract surgery only

Twenty eyes from 20 Caucasian patients (11 males and 9 females) were included (Table 1). The BCVA increased significantly after surgery (*p* < 0.001, paired *t*-test). Mean CECD using semiautomatic counting was 2528 ± 383 preoperatively and 2065 ± 434 postoperatively, and this decrease was statistically significant (*p* < 0.001, paired *t*-test). The mean percentage of cell loss was 17.9 ± 15.1%. The COV did not increase significantly after surgery (*p* = 0.079, paired *t*-test), but the hexagonality percentage decreased significantly after surgery (*p* = 0.005, paired *t*-test).

### Comparison between both studies

There were no significant differences between the age and gender composition of the two studies (Table 1). Baseline BCVA at baseline and 1 month after surgery was 0.49 ± 0.21 and 0.98 ± 0.04, respectively, and when compared between the two studies, the baseline was similar (*p* = 0.25, Kruskal–Wallis), but the postoperative was higher in the phacoemulsification group (*p* = 0.017, Kruskal–Wallis).

Baseline CECD was similar in both studies but there were significant differences between the densities found 1 month after surgery in the different groups (Table 1). Mean percentages of cell loss 1 month after surgery were 2.4%, 17.9%, 30.7% and 43.6%, after trabeculectomy, phacoemulsification and one-step or two-step phacotrabeculectomy, respectively (Table 1). At this time, we found significant differences between the phacoemulsification and the two-step phacotrabeculectomy groups (Tukey, *p* < 0.0003).

The COV and the percentage of hexagonality were similar between the groups of both studies at baseline (Table 1), but, 1 month after surgery, the COV was significantly higher in the phacoemulsification alone group and the percentage of hexagonality was significantly lower in the two-step phacotrabeculectomy group.

## Discussion

The objective of this study was to analyse the effects on the corneal endothelium of trabeculectomy, one- or two-step phacotrabeculectomy or phacoemulsification. For this purpose, we included two prospective studies.

### Short- and long-term prospective study: phacotrabeculectomy in one or two steps

In the first prospective study, we included 40 eyes of 38 Caucasian patients that were followed 12 (one step) or 15 months (two step; 3 months after trabeculectomy + 12 months after phacoemulsification). Mean age and sex of patients, glaucoma diagnosis and preoperative and final BCVA were also similar in both groups.

Preoperative IOP decreased significantly in both groups, 50% after the one-step procedure and 54% after trabeculectomy in the two-step procedure and remained stable. Other studies have reported decreases between 30 and 60% after combined phacotrabeculectomy [12, 22–24] and between 35 and 65% after trabeculectomy [10]. We did not find an elevation of the IOP after phacoemulsification in the two-step group, as found by other authors [25].

Baseline CECD was similar to those reported by other authors [9, 12, 18, 26]. CECD decreased significantly immediately after one-step phacotrabeculectomy, remained stable thereafter and decreased 31.6% by 12 months after surgery. In our previous study, we had found a decrease of 24.85% [14]. Other authors have documented decreases of only 7–16% after one-step phacotrabeculectomy [12, 17, 18, 27, 28], but some reported 20–24% decreases [29] closer to ours. We use a two-site approach that may cause more endothelial cell loss than the one site [11, 17].

In the two-step group, CECD decreased non-significantly (3.2%) after trabeculectomy. Other studies have reported CECD decreases between 2 and 10% [7, 8, 10, 11, 14, 28, 29], and it may be higher with mitomycin C application [13, 15, 16, 30]. In our previous study, we had documented a non-significant loss (6.35%), but neither in this nor in the previous study did we use mitomycin C.

In the two-step surgery group, we observed a large drop of the CECD after cataract surgery that amounted 41.8% at 1 month and 42.6% at 12 months. CECD was significantly lower in this two-step group than in the one-step group, at all the postoperative intervals, and thus the two-step procedure causes significantly more endothelial cell loss. In our previous retrospective study, we had found similar percentages of cell loss [14], and this study confirms our previous finding. To our knowledge, this is the first prospective randomised study that analyses corneal endothelial cell loss after two-step cataract phacotrabeculectomy.

The baseline COV were similar in the one-step and two-step groups, also similar to those found in our previous study [14] and did not decrease significantly after phacotrabeculectomy. Most studies have documented COV between 30 and 40%, but also values of up to 60% [3, 10, 14, 31], and increasing values when there is endothelial loss [10, 11, 14, 18, 32]. Neither in this study nor in our previous study [14] have we found significant COV increases after surgery, in accordance with the results of other authors [10, 11]. Thus, the COV is not a very good indicator of corneal endothelial cell loss and this has been also suggested by other authors [31, 32].

The baseline percentage of hexagonality was similar in the one- and two-step groups. Other authors have reported large interindividual variations (between 45.1 and 80%) and decreases with endothelial cell damage [3]. We did not find significant changes of this percentage after one-step phacotrabeculectomy or after trabeculectomy, but we found a significant decrease after two-step phacotrabeculectomy. Our previous study [14] and other studies have not found significant decreases of this percentage after trabeculectomy [10, 11] or after phacotrabeculectomy [11, 28].

### Short-term prospective study: phacoemulsification alone and comparison of the results from both studies

The age and gender composition of the phacoemulsification only group by was similar to the groups of the phacotrabeculectomy study. Baseline CECD was also similar to the observed in the phacotrabeculectomy study groups and decreased significantly after phacoemulsification. Mean percentages of corneal endothelial cell loss 1 month after phacoemulsification were 17.9% with the semiautomatic counting method. Although CECD 1 month after phacoemulsification were lower than after trabeculectomy and higher than after phacotrabeculectomy, it showed only significant differences with the two-step phacotrabeculectomy group (Table 1). The reported percentages of endothelial cell loss after cataract surgery range between 7 and 26% [1, 9, 33–38] and are related to: age, surgical technique, lens hardness, ultrasound time, ACD and complication [37–39]. As in the phacotrabeculectomy study, after phacoemulsification, the COV did not increase significantly but the percentage of hexagonality decreased significantly. Thus, both our studies indicate that the percentage of hexagonality is a better indicator of corneal endothelial cell damage.

### Confoscan 4™ confocal microscopy and conclusions

We find important CECD decreases after phacoemulsification and after phacotrabeculectomy, generally higher than those reported previously by other authors, and we think that it may be due to difference in the methods used. Other authors use non-contact or contact specular microscopy and also automatic cell counting, while we use Confoscan 4™ confocal microscopy and semiautomatic cell counting in four images and we think that our method is more accurate and better reflects cell loss. Automatic endothelial cell counting may overestimate cell density, especially with lower cell densities [40, 41] because the numbers of cells included in the analysis decrease and influence the results [32, 42–44].

When glaucoma and cataract coexist, the patients need early improvement in visual acuity and phacoemulsification and glaucoma surgery should be performed. There are many surgical techniques for glaucoma that range from minimally invasive glaucoma surgery to cyclophotocoagulation. However, for many authors, one or two-step phacotrabeculectomy is still the procedure of choice. In this prospective study, we have found CECD decreases of only 3.2% after trabeculectomy, 17.9% after phacoemulsification and 31.6% and 42.6% after one-step or two-step phacotrabeculectomy, respectively. We conclude that the two-step procedure, in which we perform trabeculectomy and 3 months later phacoemulsification, causes more endothelial cell loss than the one-step procedure and this result confirms the results our previous retrospective study [14]. We think that the increased cell damage found after the two-step procedure may be due to the cumulative effects of two surgical traumas or to a negative conditioning lesion effect of the first surgery on the corneal endothelium.

## Summary

### What was known

- Anterior segment surgery decreases corneal endotelial cell density and thus it is important to know the amount of cell loss caused by the different surgical procedures.
- Trabeculectomy causes less corneal endotelial cell loss than phacoemulsification, but combined phacotrabeculectomy may cause more cell loss than phacoemulsification alone.
- When glaucoma and cataract coexist, the surgical procedure of choice for most authors is phacotrabeculectomy that can be performed in one or two steps.
- In a previous retrospective study we documented that 2-step phacotrabeculectomy (trabeculectomy followed 3 months later by phacoemulsification) causes more corneal endotelial cell loss than 1-step phacotrabeculectomy.

### What this paper adds

- This prospective randomized study analyzes the effects of trabeculectomy, phacoemulsification and phacotrabeculectomy on corneal endotelial cell loss. This is the first prospective randomized study that compares the effects of 1- or 2-step phacotrabeculectomy on cell loss.
- For this purpose, we have used reliable methods: Confoscan 4™ and semiautomatic quantification of cell densities.
- We document that trabeculectomy causes less cell loss than phacoemulsification, phacoemulsification less cell loss than phacotrabeculectomy and 1-step phacotrabeculectomy less cell loss than 2-step phacotrabeculectomy.
